# Risk factors for neonatal early-onset group B streptococcus-related diseases after the implementation of a universal screening program in Taiwan

**DOI:** 10.1186/s12889-018-5358-0

**Published:** 2018-04-02

**Authors:** Li-Chen Hung, Pei-Tseng Kung, Tsan-Hung Chiu, Hsun-Pi Su, Ming Ho, Hui-Fen Kao, Li-Ting Chiu, Kuang-Hua Huang, Wen-Chen Tsai

**Affiliations:** 1 Department of Public Health, China Medical University, Taichung, Taiwan, Republic of China; 2 Department of Healthcare Management, Yuanpei University of Medical Technology, Hsinchu, Taiwan, Republic of China; 3 Department of Healthcare Administration, Asia University, Taichung, Taiwan, Republic of China; 4 Department of Medical Research, China Medical University Hospital, China Medical University, Taichung, Taiwan, Republic of China; 5 Department of Obstetrics and Gynecology, China Medical University Hospital, China Medical University, Taichung, Taiwan, Republic of China; 6 Department of Dental Hygiene, China Medical University, Taichung, Taiwan, Republic of China; 7 Department of Obstetrics and Gynecology, Asia University Hospital, Taichung, Taiwan, Republic of China; 8 Department of Health Services Administration, China Medical University, 91 Hsueh-Shih Road, Taichung, Taiwan, Republic of China 40402

**Keywords:** Group B streptococcus, Early-onset GBS-related disease, Universal screening, Preterm birth, Newborn

## Abstract

**Background:**

We examined the risk for Group B streptococcus (GBS)-related diseases in newborns born to mothers who participated in a universal GBS screening program and to determine whether differences are observed in factors affecting the morbidity for neonatal early-onset GBS-related diseases.

**Methods:**

This is a retrospective study and the study subjects were women who had undergone GBS screening and who gave birth naturally and their newborns between April 15, 2012 and December 31, 2013. Data from the GBS screening system database and the National Health Insurance database were collected to calculate the GBS prevalence in pregnant women and morbidity of newborns with early-onset GBS-related diseases.

**Results:**

The GBS prevalence in pregnant women who gave birth naturally was 19.58%. The rate of early-onset infection caused by GBS in newborns decreased from the original 0.1% to 0.02%, a decrease of as high as 80%. After the implementation of the universal GBS screening program, only three factors, including positive GBS screening result (OR = 2.84), CCI (OR = 2.45), and preterm birth (OR = 4.81) affected the morbidity for neonatal early-onset GBS-related diseases, whereas other factors had no significant impact.

**Conclusion:**

The implementation of the universal GBS screening program decreased the infection rate of neonatal early-onset GBS diseases. The effects of socioeconomic factors and high-risk pregnancy on early-onset GBS infections were weakened.

## Background

Group B streptococcus (GBS) disease is the most common infectious disease in newborns in the first week of life. The morbidity and mortality of newborns infected with GBS are significantly higher than those of normal newborns, and GBS disease is an important factor affecting neonatal sepsis, meningitis, and pneumonia [1–4]. In Taiwan, the prevalence of pregnant women carrying GBS in their birth canal is approximately 20%; the neonatal morbidity rate for GBS-related diseases is approximately 0.1%; the neonatal mortality rate for GBS-related diseases is between 10% and 13%; and the prevalence of neurological sequelae due to GBS infection is 15%, which demand high social cost and long-term medical care [5]. In view of this, identification of the key factors affecting the morbidity for GBS-related diseases as well as early response and prevention is indeed an important issue that needs to be addressed.

With regard to factors related to the morbidity of newborns with GBS-related diseases, past studies suggested that these factors included vaginal birth, maternal vaginal and rectal GBS infection, preterm babies delivered before 37 weeks of gestation, rupture of the amniotic sac > 18 h during labor, maternal temperature > 38 °C during labor, previous infant of the pregnant woman being infected with GBS, GBS detected in the urine of pregnant woman, and African or Latin American race, all of which could increase the neonatal morbidity rate for GBS-related diseases [6–12]. In addition, hospital types and levels, maternal disease history, newborn birth weight, number of fetuses, and comorbidities/complications also affect the neonatal morbidity for GBS-related diseases [13].

The implementation of all comprehensive screening policies can help understand the status of pregnant women with GBS infection. This will facilitate early detection and antibiotic treatment that will decrease the probability of neonatal infection, thereby reducing damage, regrets, and medical expenditure [1]. The incidence of neonatal GBS-related diseases can be decreased through the administration of antibiotic prophylaxis during childbirth in pregnant women who are carriers. Healthcare workers can use screen- or risk-based methods to identify pregnant women who must receive antibiotic prophylaxis [14, 15]. A screen-based method can identify GBS-positive women who must be given antibiotic prophylaxis to decrease the probability of newborns developing early-onset GBS-related diseases. However, if only a risk-based perspective is used to identify pregnant women who must receive antibiotic prophylaxis, this may result in the misclassification of women who must receive the treatment [16]. A comprehensive prenatal screening for GBS is an important health measure. Based on previous experience on the implementation of a comprehensive GBS screening in the US, promotion and execution are challenging tasks [17, 18]. In addition, the establishment of a notification system for long-term data collection can be an important reference for the government in the establishment of GBS prevention policies [19].

Among current internationally adopted strategies for preventing the neonatal early-onset GBS infection, GBS screening in pregnant women and intrapartum antibiotic prophylaxis are considered the mainstream strategies [5]. For example, the implementation of a universal GBS screening program for pregnant women has been conducted in the United States, and as a result, the rate of neonatal early-onset infection caused by GBS decreased from 1.8‰ to 0.5‰, a decrease of as much as 78%, whereas the neonatal mortality rate for GBS-related diseases decreased from 50% in 1970 to 4% in 1990, indicating that the implementation was significantly effective [10]. Based on this, the Taiwan Health Promotion Administration has begun a universal GBS screening subsidy program for pregnant women since April 15, 2012, which provides free GBS screening service for women who are in weeks 35–37 of gestation [5].

Taiwan implements the National Health Insurance, and the policy of a free universal GBS screening service was implemented in 2012. We focused on several issues, including the following under this health policy mode: whether differences in the morbidity rate for GBS-related diseases in pregnant women and newborns are observed, whether different factors affect the morbidity for neonatal GBS-related diseases, and which influencing factors are weakened or strengthened after the implementation of this universal screening program.

## Methods

### Study subjects

Our study subjects were women who had delivered naturally and who underwent GBS screening as well as their newborns after the implementation of the universal GBS screening program (between April 15, 2012 and December 31, 2013). The medical data of subjects were traced back to January 1, 2005. This study excluded women who had cesarean birth and only included women who delivered naturally as study subjects, and the scope of the discussion was restricted to neonatal early-onset GBS-related diseases.

### Data sources

This is a retrospective study, wherein secondary data analyses were performed. In addition to the GBS screening data provided by Health Promotion Administration, 2005–2013 National Health Insurance data, birth certificate application records, information on the accreditation profile of medical facilities, and household register information were also included to facilitate the linking of the information of pregnant women to that of newborns.

### Variable definition

In our study, the relevant variables consist of the maternal characteristics (age, educational level, nationality), environmental factor (degree of urbanization of residence areas), maternal economic characteristics (monthly salary), maternal health (severity of comorbidity), level and ownership of medical institutions, maternal production (whether premature rupture of membrane), infant birth (whether premature birth, birth weight), previous infant health (whether infected with GBS-related diseases).

The urbanization level of residence areas was divided into seven levels, with level 1 as the highest degree of urbanization, and level 7 as the lowest degree of urbanization. To simplify the comparisons, level 1 and 2 were combined as the reference, and the remaining 5 levels were grouped into 1 group (level 3, 4, 5, 6 and 7). The severity of comorbidity was presented as Charlson comorbidity index (CCI) revised by Deyo et al. [20].

Early-onset GBS-related diseases in this study was neonatal sepsis, meningitis, or pneumonia resulting from GBS infection, and the definition of actual infection with early-onset GBS-related disease was the primary diagnosis of the aforementioned diseases indicated by their ICD-9 codes from the hospital record for at least once within seven days of birth. International Classification of Diseases (ICD)-9 diagnosis code 038.0 was for neonatal sepsis resulting from GBS infection (Streptococcal septicemia); ICD-9 diagnosis code 320.2 was for neonatal meningitis caused by GBS infection (Streptococcal meningitis); and ICD-9 diagnosis code 482.30 (Streptococcal pneumonia NOS) and 482.32 (Pneumonia streptococcus b.) were for pneumonia caused by GBS infection. Infants delivered before 37 weeks of gestation were considered preterm, which was inferred from the diagnosis codes 644, 640.1, 640.81, and 640.91 in our study. Premature rupture of membrane (PROM) refers to a phenomenon of natural membrane rupture to allow amniotic fluid to leak out, which occurs one hour before the rhythmic pain of labor begins after 37 weeks of gestation. In our study, the main diagnosis code 658.1× and 761.1 indicated PROM.

### Statistical analysis

First, the GBS screening data were merged with the National Health Insurance data to calculate the GBS prevalence in pregnant women and the morbidity of newborns with early-onset GBS-related diseases. Subsequently, pregnant women who underwent GBS screening and delivered naturally and their newborns were selected, and the results of the GBS screening were presented as counts and percentages. In addition, the distribution of pregnant women and newborns with early-onset GBS-related diseases was also analyzed. A chi-square test was first performed to analyze the differences in morbidity rate for early-onset GBS-related diseases in newborns for each variable to identify key factors affecting the morbidity for GBS-related diseases. Subsequently, a logistic regression analysis was performed, wherein neonatal early-onset GBS-related disease was a dependent variable, and maternal characteristics, environmental factors, maternal health, characteristics of maternal medical center, maternal condition during childbirth, condition of the newborn during birth, and health condition of previous infant(s) were independent variables for the identification of factors affecting the morbidity for neonatal early-onset GBS-related diseases. Our study utilized a full model for analysis to control all variables. Aside from a few cells that contain zero values, which show that some variables were not included (maternal nationality, aboriginal, and previous infant(s) with GBS-related diseases), all other variables were inputted into the model for control. All statistical analyses were performed using SAS software (Version 9.4, SAS Institute Inc., Cary, NC).

## Results

A total of 154,088 pregnant women underwent GBS screening and delivered naturally. Among them, 30,176 had a positive screening result, with a GBS prevalence of 19.58% (Table 1). In terms of morbidity risk, the percentage of newborns contracting early-onset GBS-related diseases (including sepsis, meningitis, and pneumonia) and delivered by women who underwent screening and delivered naturally was 0.02%. Of these, the neonatal morbidity rate for GBS-positive mothers was 0.03%, whereas that for GBS-negative mothers was 0.01%, and the difference was statistically significant.

**Table 1:** Bivariate analysis of the relationship between each variable and the morbidity for neonatal early-onset group B streptococcus-related diseases

			No GBS-related disease		GBS-related diseases		
	n	%	n	%	n	%	*p*-value
Total	154,088	100.00	154,064	99.98	24	0.02	
GBS screening result							0.016
GBS negative	123,912	80.42	123,898	99.99	14	0.01	
GBS positive	30,176	19.58	30,166	99.97	10	0.03	
Maternal age							0.132
< 35 years	122,290	79.36	122,274	99.99	16	0.01	
≧35 years	31,798	20.64	31,790	99.97	8	0.03	
Mean age (Mean ± SD)	30.89	4.51	30.89	4.51	31.83	4.18	0.308^a^
Maternal nationality							NV
Taiwan	151,899	98.58	151,875	99.98	24	0.02	
Foreign	2189	1.42	2189	100.00	0	0.00	
Maternal education							0.934
High school and below	46,955	30.47	46,947	99.98	8	0.02	
College and above	107,133	69.53	107,117	99.99	16	0.01	
Monthly salary(NTD)							0.399
≦28,800^b^	93,227	60.50	93,215	99.99	12	0.01	
≧28,801	60,861	39.50	60,849	99.98	12	0.02	
Degree of urbanization							NV
1	32,981	21.40	32,981	100.00	0	0.00	
2	48,994	31.80	48,986	99.98	8	0.02	
3~ 7	72,113	46.80	72,097	99.98	16	0.02	
Aboriginal							NV
No	149,288	96.88	149,264	99.98	24	0.02	
Yes	4800	3.12	4800	100.00	0	0.00	
CCI^c^							0.035
0	133,218	86.46	133,201	99.99	17	0.01	
≧1	20,870	13.54	20,863	99.97	7	0.03	
PROM^d^							0.056
No	148,433	96.33	148,412	99.99	21	0.01	
Yes	5655	3.67	5652	99.95	3	0.05	
Preterm birth							0.024
No	150,077	97.40	150,056	99.99	21	0.01	
Yes	4011	2.60	4008	99.93	3	0.07	
Infant birth weight							0.597
<2,500	6534	4.24	6533	99.98	1	0.02	
2,500-3,499	126426	82.05	126408	99.99	18	0.01	
≧3,500	21128	13.71	21123	99.98	5	0.02	
Previous infant(s) with GBS-related diseases							NV
No	154072	99.99	154048	99.98	24	0.02	
Yes	16	0.01	16	100.00	0	0.00	
Level of medical institution							0.749
Medical center	26052	16.91	26046	99.98	6	0.02	
Regional hospital	43383	28.15	43377	99.99	6	0.01	
District hospital	39213	25.45	39208	99.99	5	0.01	
Primary clinic	45440	29.49	45433	99.98	7	0.02	
Ownership of medical institution							1.000
Public	15003	9.74	15001	99.99	2	0.01	
Private	139085	90.26	139063	99.98	22	0.02	

^a^t-test

^b^Including low-income households

^c^*CCI* Charlson comorbidity index

^d^*PROM* Premature rupture of membrane

As shown in Table 1, pregnant women who lived in a city with a high degree of urbanization delivered newborns with a 0% morbidity rate for early-onset GBS-related diseases, which was significantly lower than the morbidity rate of 0.02% for cities with a medium degree of urbanization and for other regions. For pregnant women who had the CCI score ≧ 1, the morbidity rate for neonatal early-onset GBS-related diseases was 0.03%, which was significantly higher than the 0.01% morbidity rate for those who had a score of 0. For those who had PROM, the morbidity rate for neonatal early-onset GBS-related disease was 0.05%, which was significantly higher than the 0.02% morbidity rate for those without PROM. Preterm infants had a 0.07% morbidity rate for early-onset GBS-related diseases, which was significantly higher than the 0.01% morbidity rate of full-term infants. Other variables did not show a statistically significant correlation with the morbidity rate for neonatal early-onset GBS-related diseases. Summarizing the results of the above chi-square analysis showed that variables, including GBS screening result, degree of urbanization, maternal CCI score, and preterm infant, were the main factors affecting the morbidity rate for neonatal early-onset GBS-related diseases.

The results of chi-square analysis shown in Table 1 indicated that four variables, including GBS screening result, degree of urbanization, maternal CCI score, and preterm infant, showed a statistically significant correlation with the morbidity for early-onset GBS-related diseases. Therefore, we further performed the logistic regression analysis to identify key factors affecting the morbidity for neonatal early-onset GBS-related diseases. The analysis results of the unadjusted model (Table 2) showed that four variables, including GBS screening result (unadjusted OR = 2.97, *p* < 0.05), CCI score (unadjusted OR = 2.58, p < 0.05), PROM (unadjusted OR = 3.98, p < 0.05), and preterm infant (unadjusted OR = 5.15, p < 0.05), significantly affected the morbidity for neonatal early-onset GBS-related diseases. After controlling for other variables, the adjusted model A showed that only three variables, including positive GBS screening result (adjusted OR = 2.84, p < 0.05), CCI score (adjusted OR = 2.45, p < 0.05), and preterm infant (adjusted OR = 4.81, p < 0.05), significantly affected the morbidity for neonatal early-onset GBS-related diseases, whereas other variables had no significant impact. It is worth noting that the significant effects (unadjusted OR = 3.98, p < 0.05) became insignificant (adjusted OR = 3.42, *p* > 0.05) after correcting the effects of premature rupture of membranes (PROM). This shows that the effect of PROM was reduced after considering other variables.

**Table 2:** Factors affecting the morbidity for neonatal early-onset group B streptococcus-related diseases

	Unadjusted model				Adjusted model A			
	OR	95% CI		*p*-value	OR	95% CI		*p*-value
GBS screening result								
GBS negative	1.00	–	–	–	1.00	–	–	–
GBS positive	2.97	1.32	6.69	0.009	2.84	1.26	6.41	0.012
Maternal age								
< 35 years	1.00	–	–	–	1.00	–	–	–
≧35 years	2.13	0.91	4.98	0.081	1.76	0.74	4.20	0.201
Maternal education								
High school and below	1.00	–	–	–	1.00	–	–	–
College and above	0.97	0.42	2.27	0.948	0.77	0.30	1.94	0.575
Monthly salary(NTD)								
≦28,800	1.00	–	–	–	1.00	–	–	–
≧28,801	1.72	0.77	3.82	0.185	1.68	0.70	4.05	0.246
Degree of urbanization								
1 + 2	1.00	–	–	–	1.00	–	–	–
3 + 4 + 5 + 6 + 7	1.36	0.58	3.18	0.479	1.61	0.67	3.85	0.289
CCI								
0 point	1.00	–	–	–	1.00	–	–	–
≧1 point	2.58	1.07	6.22	0.035	2.45	1.01	5.93	0.048
PROM								
No	1.00	–	–	–	1.00	–	–	–
Yes	3.98	1.19	13.34	0.025	3.42	0.95	12.31	0.060
Preterm birth								
No	1.00	–	–	–	1.00	–	–	–
Yes	5.15	1.54	17.27	0.008	4.81	1.31	17.70	0.018
Infant birth weight (g)								
< 2500	1.00	–	–	–	1.00	–	–	–
2500–3499	0.95	0.13	7.09	0.631	1.82	0.22	15.04	0.976
≧3500	1.59	0.19	13.61	0.472	3.18	0.33	30.82	0.229
Level of medical institution								
Medical center	1.00	–	–	–	1.00	–	–	–
Regional hospital	0.47	0.15	1.47	0.559	0.50	0.16	1.58	0.445
District hospital	0.43	0.13	1.40	0.405	0.50	0.15	1.69	0.471
Primary clinic	0.57	0.19	1.70	0.955	0.75	0.23	2.44	0.726
Ownership of medical institution								
Public	1.00	–	–	–	1.00	–	–	–
Private	1.05	0.25	4.45	0.951	1.24	0.28	5.57	0.779

## Discussion

In the first year after the implementation of the universal GBS screening program in Taiwan, the GBS prevalence in pregnant women who delivered naturally was 19.58%, which was not significantly different from the 20% prevalence rate before implementation [5]. In Taiwan, the GBS prevalence in pregnant women was approximately 20%, slightly higher than the global average of 17.9%. The GBS prevalence in Taiwan was similar to the 19.7% and 19.0% prevalence rates in the US and Europe, respectively, which were lower than the 22.4% prevalence rate in Africa, and higher than the 11.1%, 13.3%, and 16.7% prevalence rates in South Asia, Western Pacific, and Eastern Mediterranean, respectively [21]. In addition to factors such as age, obesity, number of childbirth, genetics, and socioeconomic status, maternal GBS prevalence was also affected by ethnicity [22–24]. The purpose of the universal screening program was to detect pregnant GBS-carrying women early and to provide prompt preventive treatment. However, it did not directly help decrease the GBS prevalence in pregnant women.

In addition, the morbidity rate for early-onset infections caused by GBS decreased from the original 0.1% to 0.02%, with a decrease of as high as 80%, indicating that after the implementation of the universal screening policy in Taiwan, the rate for neonatal early-onset infection showed a significant downward trend due to the early detection of pregnant women carrying GBS and the intervention of preventive treatment. In addition to the implementation of the universal maternal GBS screening program in the United States where a decrease of as much as 78% in the rate of neonatal early-onset infection caused by GBS was observed [10], Taiwan’s experience further verified the contribution of the universal screening policy to the decreased risk for neonatal early-onset GBS-related diseases.

With regard to factors affecting the morbidity for neonatal early-onset GBS-related diseases, only three remaining factors, including positive GBS screening result (OR = 2.84), CCI (OR = 2.45), and preterm birth (OR = 4.81) significantly affected the morbidity for neonatal early-onset GBS-related diseases after the implementation of the universal GBS screening program in Taiwan. With regard to PROM, the adjusted OR was 3.42 (*p* > 0.05), which was lower than the original value of 3.98 (*p* < 0.05), suggesting that the impact of PROM on the morbidity for neonatal early-onset GBS-related diseases became insignificant after controlling for other factors.

Preterm birth increases the chances of neonatal early-onset GBS-related diseases, and this correlation has been suggested in many works of literature [9–12, 19, 24, 25]. Thus, how to decrease preterm birth will be an important issue. The continuous tracking of the condition of pregnant GBS-infected women and antibiotic treatment can reduce the chances of preterm birth [26]. For women who have preterm birth before 37 weeks, including those who do not have prenatal GBS culture, whose culture results are unavailable, or whose culture results are undetermined, intrapartum antibiotic prophylaxis is still needed. As recommended by pharmacokinetic and microbiological evidence, for women who are admitted to the hospital due to precipitate labor and for those who delivered in > 4 h, a complete course of antibiotic treatment cannot be given; however, a minimum of two-hour antibiotic treatment can still provide protection for newborns [21].

Because Taiwan implements National Health Insurance, people’s accessibility to medical care has increased geographically and economically. Therefore, disease morbidity of newborns does not change due to different degrees of urbanization in the area of residence or socioeconomic status (maternal education level, monthly salary). In addition, for women with high-risk pregnancy (advanced maternal age, overweight infant), the Taiwan National Health Insurance will pay for the cost of cesarean section as long as the doctor evaluates and determines that medical necessity requirements are met. This study already excluded women who had a cesarean section, and thus, the effects of maternal age and infant birth weight on the morbidity for neonatal early-onset GBS-related diseases were not statistically significant (*p* > 0.05). In addition, under the Taiwan National Health Insurance, hospitals must pass the hospital accreditation for approval as a National Health Insurance-appointed medical institution. This general criterion also promotes the medical care of each medical institution to reach a certain quality, and therefore the neonatal morbidity rate did not vary due to types of medical institutions.

With regard to the fact that prophylactic antibiotic treatment in pregnant women still cannot completely eliminate the occurrence of neonatal early-onset GBS-related disease, further analysis of this study found that in pregnant GBS-positive women who underwent antibiotic treatment (*n* = 23,826), there was still a 0.04% chance for their newborns to acquire early-onset GBS-related diseases. Similarly, the literature also pointed out that even for women who were not in the high-risk group for GBS during pregnancy, had a negative GBS screening result, or were administered antibiotics, their newborns can still be infected with GBS-related diseases [27–29].

In addition, the method of antibiotic administration, the time of use, and the appearance of resistant strains also affect its effectiveness in preventing neonatal GBS-related diseases. Intravenous injection is the only recommended route of administration [10] because it allows the drug to maintain at a high concentration in the amniotic fluid. Pregnant women who were tested positive for GBS were administered intrapartum antibiotic prophylaxis (IAP). A continuous 48-h prophylactic antibiotic treatment produces the greatest protective effects in newborns. However, drug resistance is also a factor limiting its effectiveness due to the widespread use of antibiotics. Up to 15% of the GBS strains are resistant to clindamycin, and 7% –25% of the strains are resistant to erythromycin [30]. The issue of drug resistance also results in unsatisfactory implementation effectiveness of the preventive antibiotic treatment.

Correct screening tools are also an influencing factor that cannot be ignored. A study evaluating the GBS guideline proposed by the US Centers for Disease Control and Prevention suggested that among full-term newborns who were infected with GBS, 61.4% were born to mothers with a negative GBS screening result [31, 32]. Further analysis of this study also found that the newborns of women who had a negative GBS screening result still had a 0.01% infection rate (*n* = 123,912). Thus, reducing the false-negative rate of the screening tool and adjusting the screening procedure to increase correctness will be key factors for effective prevention of GBS-related diseases. With regard to the current level of medical care, the methods for neonatal risk assessment and disease prevention can still be improved. Therefore, the development of more sophisticated diagnostic techniques to distinguish high-risk newborns will help clinicians form appropriate treatment guidelines and preventive measures, thereby reducing the chances of neonatal early-onset GBS-related diseases [33, 34].

Based on Taiwan’s experience, the implementation of the universal GBS screening program can reduce the morbidity for neonatal early-onset GBS disease. In addition, under the National Health Insurance System, the effects of socioeconomic factors (degree of urbanization of the residential area, maternal education level, and monthly salary) and high-risk pregnancy (advanced maternal age, overweigh infant) on the neonatal early-onset GBS diseases are weakened because of increased public accessibility to medical resources and the general improvement of medical care quality. However, maternal and neonatal pathological conditions (CCI score, preterm birth) remain to be the key factors affecting neonatal early-onset GBS diseases. If a sound health management plan can be provided to pregnant women to decrease the preterm birth rate, PROM, and CCI score and a universal screening program can be implemented, then the morbidity rate for neonatal early-onset GBS-related diseases can be reduced.

With regard to the limitation of this study, the universal GBS screening policy was implemented in Taiwan since April 15, 2012, and during the course of this study, the data from the National Health Insurance database were only available through the end of 2013. Thus, only the data from April 15, 2012 to December 31, 2013 were analyzed. In addition, the data in this study were from secondary databases, and partial data of the newborns and mothers were lacking because databases could not be combined. This type of situation was excluded from calculation during analysis.

## Conclusion

The implementation of the universal GBS screening program decreased the infection rate of neonatal early-onset GBS diseases. The effects of socioeconomic factors and high-risk pregnancy on early-onset GBS infections were weakened.

## Abbreviations

CCI: Charlson Comorbidity Index
GBS: Group B streptococcus
ICD: International Classification of Diseases
PROM: Premature Rupture of Membrane
